# Robust methods for accurate diagnosis using pan-microbiological oligonucleotide microarrays

**DOI:** 10.1186/1471-2105-10-S2-S11

**Published:** 2009-02-05

**Authors:** Yang Liu, Lee Sam, Jianrong Li, Yves A Lussier

**Affiliations:** 1 Center for Biomedical Informatics and Section of Genetic Medicine, Dept. of Medicine, The University of Chicago, Chicago, 5841 South Maryland Ave, Ill, USA; 2 UC Cancer Research Center, The University of Chicago, Chicago, 5841 South Maryland Ave, Ill, USA; 3 Institute for Genomics and Systems Biology, The University of Chicago, Chicago, 5841 South Maryland Ave, Ill, USA; 4 Computation Institute, The University of Chicago, Chicago, 5841 South Maryland Ave, Ill, USA

## Abstract

**Background:**

To address the limitations of traditional virus and pathogen detection methodologies in clinical diagnosis, scientists have developed high-throughput oligonucleotide microarrays to rapidly identify infectious agents. However, objectively identifying pathogens from the complex hybridization patterns of these massively multiplexed arrays remains challenging.

**Methods:**

In this study, we conceived an automated method based on the hypergeometric distribution for identifying pathogens in multiplexed arrays and compared it to five other methods. We evaluated these metrics: 1) accurate prediction, whether the top ranked prediction(s) match the real virus(es); 2) four accuracy scores.

**Results:**

Though accurate prediction and high specificity and sensitivity can be achieved with several methods, the method based on hypergeometric distribution provides a significant advantage in term of positive predicting value with two to sixty folds the positive predicting values of other methods.

**Conclusion:**

The proposed multi-specie array analysis based on the hypergeometric distribution addresses shortcomings of previous methods by enhancing signals of positively hybridized probes.

## Background

Since their inception in the early nineties [1], microarrays have become widely used in research and are increasingly translating to clinical practice because of their low cost and high-throughput analysis of gene expression [2,3]. Indeed, expression analysis arrays have been employed to predict clinical survival [4], calculate prognosis indexes and determine molecular signatures [5,6]. SNP arrays [7] are being used to study allelic variants of disease [8]. Similarly, Comparative Genomic Hybridization arrays have been employed extensively in investigating cancer tumors [9,10]. Assuming oligo arrays can achieve high sensitivity and specificity, their application for diagnosis of an infectious agent is valuable for the following reasons: (i) the increase need for multiplexing to monitor in emerging zoonoses, (ii) rapid, unbiased, and cost effective detection of infectious diseases. Indeed, the state of the art in viral diagnosis is based on the antibody response and may take up to two weeks to be detected in the blood. In contrast, Oligonucleotide arrays measure the viral RNA and allows for early detection within hours. Additionally, a large number of acute viral infections share common clinical signs and symptoms. Furthermore, clinicians currently have to ponder the additional cost and benefits of investigating infrequent infectious for which the hospital is not equipped to assay and require significant time and effort to elucidate.

Researchers have thus developed pan-viral and pan-microbial oligonucleotide arrays [11-14] for use with clinical specimens. Among them, the GreeneChip is currently the most comprehensive multiplex array for detection infectious agents, comprising about 30,000 probes designed to detect 1710 vertebrate viruses, 135 bacteria, 73 fungal and 63 parasite genera [13]. 2007 marked the year of an important proof-of-concept: two documented case reports of multiplexed arrays detecting an infectious agent when traditional methods of investigation had failed (GreeneChip [13], and ViroChip [15]).

Analyzing accurately the data from the hybridization patterns from these arrays is of utmost clinical importance and remains challenging for the following reasons: (i) species' strains are represented by multiple probes targeted in distinct genes for increased precision, (ii) thresholds where an array probe can be considered positive or negative are somewhat arbitrary, and (iii) non specific cross-hybridization patterns can lead to false positive rates, (iv) detention of microbes must allow for identifying multiple co-infections (e.g. a simple prioritization of infectious agents is of limited value). Initially, previous studies addressed this issue by comparing the total count of hybridized probes with probe sets designed for different agents to identify the correct agent(s), but this approach was found limited in precision [11,12]. Recently, more comprehensive methods for interpreting the hybridization patterns have been developed: (i) an energy-profile based prediction program, which requires a training microarray set to train the program first [16]; (ii) a Log-transformed analysis of microarrays using p-values (GreeneLAMP) [13]; and (iii) high-density resequencing microarray using much longer length probes [17].

In this study, we provide a description and an evaluation of a statistical method we developed to accurately predict the species on a pan-virual oligonucleotide arrays.

## Results and discussion

**Figure 1:**
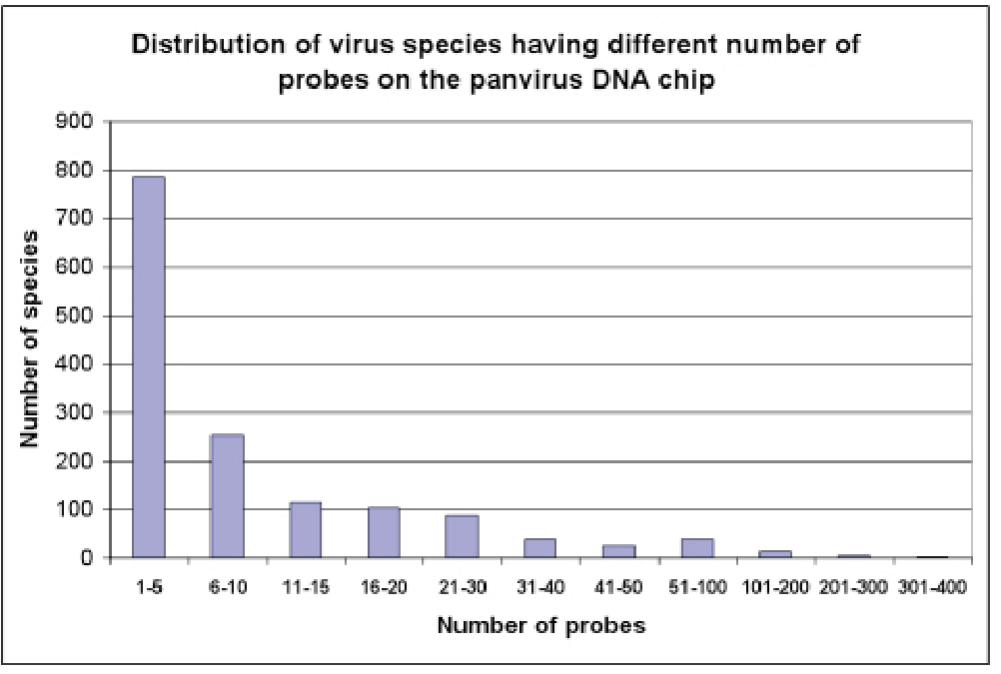
**Distribution of the number of probes per species in the GreeneChip**. Since the design of the array is based on viral strains, we design many strain specific probes in human viruses (e.g. HIV, Influenza). Indeed, Genbank often contains multiple entries of fully sequenced genomes for human viruses strains allowing for straightforward calculation of conservation and specificity. In contrast, some other vertebrate viruses may have as little as a single Gnebank entry and perhaps an incomplete genome. In the latter case, probes were designed using principles of phylogenetic protein domain conservation [24].

In this study, we evaluated six different methods over a pan-virus array (a version of the GreeneChip array [13]) designed to detect 1,710 vertebrate viruses. Although we attempted to include other methods [16,17] in the evaluation, we found several to be non-applicable to oligonucleotide microarrays [17] or impractical to implement (requiring a training dataset derived from the same DNA amplification method as the sample data) [16]. Figure 1 illustrates the distribution of the probes on the chip to each of the virus species. Some viruses such as HIV and influenza are significantly more heavily sequenced. As a result, more probes on the chip are designed to these species to cover different mutations on their DNA sequences. On the other hand, some viruses contain limited sequence information, sometimes only a partial single genomic sequence is available and are thus represented by a limited number of probes.

Each method was applied to the hybridization results composed of four West Nile virus samples. As described in the methods, three different pools of probes were selected based on the rank of the intensity of hybridizing probes, representing 0.5%, 1%, and 5% of the total probes on the chip. The virus species predicted by these methods were ranked and compared with the correct virus tested on the chip. The ranks are listed on the Supplementary T 2 . According to the ranks derived by these methods, the hypergeometric method performs the best out of the six methods and the LTRM method is only slightly behind in identifying the correct agent.

**Figure 2:**
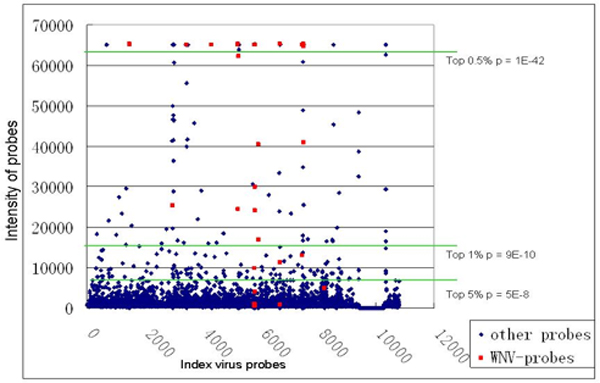
**An oligonucleotide chip hybridized by DNA of West Nile virus**. Left: significance of mapping to West Nile virus (Taxon 11082) by the hypergeometric method.

To illustrate the cutoffs of top probes in intensity at the 0.5%, 1% and 5% thresholds, we plot the distribution of the intensity of hybridized probes of a chip tested against West Nile virus chip 1 (Figure 2). The dots highlighted in red denote the probes that are designed to hybridize to the West Nile virus. Though some probes designed for the West Nile virus have the highest intensity level, some are not hybridized to the virus DNA at all. It also demonstrates that there is a certain level of cross hybridization (denoted by the blue dots). The p-values calculated using the hypergeometric method are also shown. In our preliminary results over other assays (data not shown) and in the presented assay (Tables 1 and 2), the sensitivity (recall) of every method was comparable, and specificity was high, however the LTRM had significantly better positive predicting values than all methods, with the exception of the proposed hypergeometric method outperforming the LTRM. Though other approaches can also identify the correct agent though a ranking system, the hypergeometric method is able to significantly enhance the hybridization signal of target agents and reduce the noise due to cross-hybridization.

**Table 1:** Accuracy of different methods for analyzing single instances of multiplexed microbiological arrays separately (n = 4).

	**Recall/sensitivity**	**Specificity**	Positive predicting value (precision)*	**Negative predicting value**
**Probe count**	**100% (0.0%)**	89.4% (0.5%)	1.5% (0.2%)	**100% (0.0%)**
**Probe count w/threshold**	**100% (0.0%)**	90.3% (0.4%)	1.7% (0.3%)	**100% (0.0%)**
**Probe ratio**	**100% (0.0%)**	97.7% (0.1%)	4.6% (0.4%)	**100% (0.0%)**
**Probe ratio w/threshold**	**100% (0.0%)**	98.6% (0.1%)	5.9% (1.5%)	**100% (0.0%)**
**Log transform ranking**	**100% (0.0%)**	99.8% (0.2%)	39.1% (32.2%)	**100% (0.0%)**
**Hypergeometric (proposed method)**	**100% (0.0%)**	**100% (0.0%)**	**100% (0.0%)**	**100% (0.0%)**

Average value and standard deviation for all samples are shown. Best values are bolded. * Examples of species' predictions by all methods are provided in Supplementary T 1  and clarify the positive predictive value scores below.

**Table 2:** Accuracy of different methods for analyzing repeated instances of arrays (n = 2).

	**Recall (sensitivity)**	**Specificity**	**Positive predicting value (precision)**	**Negative predicting value**
**Probe count**	**100% (0.0%)**	95.1% (1.7%)	2.7% (0.7%)	**100% (0.0%)**
**Probe count w/threshold**	**100% (0.0%)**	95.6% (1.6%)	3.0% (0.4%)	**100% (0.0%)**
**Probe ratio**	**100% (0.0%)**	98.6% (0.2%)	6.5% (1.0%)	**100% (0.0%)**
**Probe ratio w/threshold**	**100% (0.0%)**	99.3% (0.1%)	9.3% (0.7%)	**100% (0.0%)**
**Log transform ranking**	**100% (0.0%)**	99.9% (0.1%)	59.7% (9.8%)	**100% (0.0%)**
**Hypergeometric (proposed method)**	**100% (0.0%)**	**100% (0.0%)**	**100% (0.0%)**	**100% (0.0%)**

Average value and standard deviation for all samples are shown. Best values of accuracy scores are bolded in each category.

We also combined the hybridization results from chip pairs to improve predictive accuracy. Results are shown in Supplementary T 3 . Comparing the ranking and accuracy scores between the single chips and the grouped chips (Table 1 and 2), the accuracy scores for the paired chips show improvement over those than those of the single chips, especially in terms of positive predicting values. For example, the LTRM method has an increase in the positive predicting value from 39.1% to 59.7%. Overall, comparing both their ranking and accuracy scores (sensitivity and positive predicting value), the hypergeometric method performs better at the predicting positive value and is better or equivalent to other methods on other accuracy scores. Its positive predicting value is approximately 2~60 fold better than those of the other methods.

### Limitations

While the simple probe count method can, in principle, detect species with as few as one probe, the cost of confirming the predictions likely to become prohibitive due to the large number of false positives. This is demonstrated in Supplementary T 2  where selecting the 50 topmost hybridized probes may lead to dozens of false positive predicted species, and the number of false positive predictions rapidly increases with the number of probes considered. In addition, viruses with close phylogeny could also cause problems due to cross-hybridization on the chip, substantially confounding the detection process.

### Future studies

The LTRM method which also outperformed most methods is based on the log transform of expression level, while our method is based on the direct ranking of the expression levels. There is a theoretical advantage to pool the two methods together in repeated essays where the log normalization associated with the error rate could together better rank the significant probe sets than the expression level alone. We are currently exploring the joint use of the log transform and the hypergeometric distribution. The methods outlined in this study are focused on using probe-level hybridization to determine the significance of each detectable, however future studies should also address the gene level (orthologos, paralogs). Indeed, cross-hybridization of probes designed for the same genes in different strains or for orthologs in different species, could cross-hybridize when mutations affect the region in a given strain of virus even if the probes were originally designed with sufficient differences in the sequence of nucleic acid base pairs. As data becomes publicly available, we will establish the scalability of our method to these arrays as well.

## Conclusion

Our results show that, for the prediction of pathogens, high specificity and sensitivity can be achieved with a broad range of analytical methods. Only two prediction methods correctly ranked the tested pathogen in all tests, the hypergeometric and the LTRM method. To our knowledge, we conducted the first hypergeometric model of probe hybridization for interpretation of arrays with multiplexed species-probes. It differs conceptually of every other method because it provides an adjusted p-value below which no predictions are made. In contrast, previous methods do not provide an objective threshold in their stratification of species, and such threshold is established arbitrarily, leading to poor positive predicting value. As hypothesized, the method based on the hypergeometric distribution provides a significant advantage in terms of positive predicting value. When experiments are repeated with the same sample on additional arrays, the method also performs significantly better than the other methods. Beyond oligonucleotide arrays, the hypergeometric-based method is scalable and could be applied to other or newly developed multiplexed technologies for characterizing pathogens.

## Methods

We conducted analyses on the virus-only version of the pan-microbial GeeneChip that we co-developed with Dr. WI Lipkin's research group and collaborators [[13,24], Supplementary File 1 at ]. Array probes for each species's strains were computed based on principles of genetic essentiality (e.g. polymerases for viruses replication) and of conservation (e.g. protein domains conserved across related species for strains with few or only one genomic sequence available). The pan-virus chip contains a total of 9,045 60 mer probes of conserved sequences covering 1,710 vertebrate virus targets derived from an integrated DNA sequence data from 4 genomic databases, including (i) NCBI taxonomy data [18], (ii) Pfam database [19], (iii) Uniprot [20], and (iv) GenBank DNA sequences [21] (see Supplementary materials for more details ). Extensive preliminary evaluations were conducted on assays derived from infected cell cultures [data not shown]. The described evaluations are based on four independent tests on hybridization results from samples infected by the West Nile virus (Strain NY99) (4 repeats).

We apply six different methods to analyze the array hybridization patterns and calculate and compare their accuracies in detecting the correct virus DNA present in the hybridized sample. We evaluate each method assuming three different thresholds to identified positively hybridized (significant) probes. Each of the three significance levels is based on relative expression level ranking: (i) 50 probes (~0.5% of total probes, ~5–6 standard deviations (SD) from the mean probe intensity), (ii) 100 probes (~1% of total, ~3–4 SD), and (iii) 500 probes (~5% of total, ~2 SD). The methods used for species identification are:

1) Probe count: A species is considered positive if any of its probes were top positive probes. The predicted virus species are ranked by the count of positive probes.

2) Probe count with threshold: This is the same as the probe count method, with the exception that species that have less than 3 significantly hybridized probes are ignored.

3) Probe ratio: The probe ratio is calculated by dividing the number of significantly hybridized by total number of probes for each species. Species are considered positive if the ratio of mapped probes in a species is more than 30%. The predicted viruses are ranked by their ratios.

4) Probe ratio with threshold: This is the same as the probe ratio method, with the exception that species that have less than 3 significantly hybridized probes are ignored.

5) Log Transform Ranking Method (LTRM): We followed the GreenesLAMP procedure [13].

6) Hypergeometric distribution: The adjusted p value of each given species was derived. Species with a corrected p-value < 5% are considered potentially present in the specimen and ranked. The hypergeometric distribution function is described in Equation 1 [22]

(1) $$p(i>=m|N,M,n,m)=\sum_{i=m}^{n}\frac{\left(\frac{M}{i}\right)\left(\frac{N-M}{n-i}\right)}{\left(\frac{N}{n}\right)}$$

In this analysis, "N" represents the total number of probes on the chip (9,045); "M" represents the number of probes for a given species of virus; "n" represents the number of significantly hybridized probes considered, and "m" represents the probes significantly expressed in a species and the top probes.

The p-values for each species were adjusted for multiple comparisons using the Šidák function (Equation 2) [23]:

(2) *p*' = 1-(1-*p*)^*k*^

Where p' and p represent the adjusted and unadjusted p-values, respectively, and k represents the number of independent tests, which in this case is the number of virus species being tested on the chip for which at least one probe was considered significantly hybridized.

Accuracy scores were calculated for each method. In these calculations, a true positive score is established when the species known to be present in the biological specimen hybridized was predicted and ranked, regardless of the rank (note that in this series of experiments, only one species was known to be present). A false positive score is calculated by counting the number of species predicted and ranked but not known to be present in the well characterized specimen. True negative and false negative scores are also calculated at the species level, including: sensitivity (recall), positive predicting value (precision), specificity, and negative predicting value.

While a single array might be sufficient for screening; in the presence of a severe illness of patients it is likely that a biological sample would be assayed on multiple arrays in order to increase the accuracy of the diagnostic. Consequently, we evaluated the accuracy of the all the methods both as a single assay (Table 1) and also by pooling arrays together two-by-two (union of significant probes): (i) West Nile virus chips 1 and 2; (ii) West Nile virus chips 3 and 4 (Table 2). Accuracy scores were then calculated for each group following the same procedure described above. The 50,100 and 500 probes were selected from each chip and the common top probes in each group were identified.

Experiments were conducted over Sun Solaris Server and took less than a minute to compute. Methods were implemented in Perl and are available upon request.

## Competing interests

The authors have no direct financial interest in the analysis of panmicrobial arrays (this work). The authors acknowledge a related interest: they have submitted an invention report to Columbia University on the design of pan-microbial arrays.

## Authors' contributions

Yang Liu, Lee Sam and Jianrong Li implemented distinct components of the analysis of preliminary interpretation of results. Yang Liu and Yves Lussier conceived the analytical method and, the discussion and the conclusion. Yves Lussier managed the overall research.
